# Discovery of a New Species of Inguinal Hernia

**Published:** 1830-01-01

**Authors:** 


### HOPITAL BEAUJON.

### Discovery of a New Species of Inguinal Hernia.

Such is the heading of a case, such the burthen of many preliminary observations from the reporter, in the Journal Hebdomadaire.* Our readers no doubt will stare at the announcement, after all the laborious dissections and laboured descriptions that already conspire to bother the brains of our young aspirants to collegiate diplomas. But so it is, and it only remains to narrate the case that is thus destined to swell the present dread array of minutiæ in the history of hernia.

The subject, to our honour be it spoken, was an English groom, ætatis 27, admitted into hospital on the 1st of June, with an oblong tumour, the size of a couple of fists, extending from the left inguinal ring to the bottom of the scrotum. The skin was red and tense; pressure was painful and exasperated a fit of colic which distressed the patient; constipation; vomiting of bilious matter; small and frequent pulse; cold moist skin. He had been subject for five years to a hernial tumour in the groin, which would seem to have never entirely returned, and for which he wore no truss. On the 31st of May, after violent exertion, he experienced sharp pain in the tumour, which soon became hard, enlarged, and painful to the touch, and colic and nausea quickly succeeded. Between the first occurrence of these symptoms and his admission attempts at reduction had been made without success.

The warm bath twice repeated, bleedings from the arm, and two tobacco enemata were prescribed, but no amendment was found on the 2nd, and the operation was performed. The sac contained an enormous mass of omentum, with a loop of intestine six or eight inches in length at its posterior part. The omentum was sound, the intestine of port wine colour, but elastic, firm, and covered with lymph. The stricture was divided directly upwards, but on trying to reduce the gut, it returned as if by a rebound, and this part of the operation was only concluded after tedious, difficult and painful efforts. What was now to be done with the omentum? excise it, or leave it where it was? M. Blandin determined on the latter, and after a proper dressing, an enema, and a bleeding, the business was at length completed.

No stool, however, succeeded, (a primâ facie proof that a stricture remained) and on the 3d, we find the patient worse in every respect. The omentum now presented blackish-brown patches, and the belly was the seat of pain. A castor oil enema, thirty leeches to the abdomen, and emollient fomentations were tried in vain, for the patient continued to sink and died that afternoon.

*Dissection on the morning of the 5th.*—Peritoneal inflammation, with sero-albuminous effusion into the abdomen—almost the whole floating portion of the omentum in the sac; and the transverse arch of the colon dragged down, and held in contact with the abdominal parietes in the groin. On opening the inguinal canal from above, and drawing out the omentum, the intestine that had been thought to be returned, was found lying in the canal itself, and occupying a *cul-de-sac*, situated at its upper and internal part, and "formed by the hernial sac depressed on this side." In endeavouring to account for this appearance, it was quickly perceived that the protruded parts had passed out by a laceration (*éraillement)* of the fascia transversalis, situated about two lines behind and above the internal ring, or superior orifice of the inguinal passage; that having passed through this laceration they had then become lodged in the canal, and had extended both downwards to the scrotum, and upwards to the internal ring, producing by this double course a double depression or cul-de-sac in their peritoneal envelope. This disposition of the hernia explained the difficulty of reduction, as the gut during these efforts instead of passing back through the slit in the fascia, was thrust up into the summit of the inguinal passage. It also seems to us that the laceration itself through which the rupture took place could not have been divided to any extent, if at all, or the parts would still have been returned, though probably not with such ease as under ordinary circumstances.

It is evident that the above was only a species of *direct* hernia after all; not occurring, as usually happens, in the weak part opposite the outer ring, or in some other portion of the abdominal parietes, where nature or accident opens the way, but *into* the inguinal canal. Of course, there is no reason why this description of hernia, always an anormal occurrence at the best, should not take place here as well as elsewhere; but still we are not aware of any authentic or specific cases of the kind, except the present.

---

### XVII.

### BATH UNITED HOSPITAL.

[Mr. BURY Reporter.]

